# Effects of Pull-Up Training on 50-Meter Freestyle Swimming Performance: A Preliminary Analysis

**DOI:** 10.7759/cureus.65397

**Published:** 2024-07-25

**Authors:** Carlota Pardo-Atarés, Eduardo Generelo, Rafel Cirer-Sastre, Isaac López-Laval, Sebastian Sitko

**Affiliations:** 1 Faculty of Health and Sport Science, University of Zaragoza, Huesca, ESP; 2 National Institute for Physical Education of Catalonia, University of Lleida, Lleida, ESP

**Keywords:** swimming training, upper body strength, sprint performance, freestyle, pull-ups

## Abstract

This study examined the impact of incorporating pull-up exercises into the training routines of competitive swimmers on their performance outcomes. Eight swimmers (average age 21 ± 6.7 years, height 178 ± 5.3 cm, body mass 73 ± 7.0 kg) were selected and participated in a 10-week intervention, during which pull-up exercises were added to their regular strength training sessions. Performance was evaluated through tests measuring the sprint start speed and 50-meter freestyle swim times before and after the intervention. Statistical analyses showed significant improvements in swimming explosive strength, as measured by a 15-meter time trial (p = 0.014). In addition, resistance to explosive strength, as indicated by total time in a 50-meter time trial, improved significantly (p = 0.008), particularly in the first 25 meters (p = 0.014), although not in the second 25 meters (p = 0.078). These findings highlight the critical role of upper body strength and power in enhancing swimming performance, especially in sprint events. In conclusion, incorporating pull-up exercises into training regimens appears promising for improving upper body strength and power among swimmers. Future research should address the study's limitations by using larger, more homogeneous samples and more rigorously controlling variables such as age, gender, and training schedules. These efforts could provide clearer insights into the effectiveness of pull-up exercises in optimizing swimming performance, particularly in sprint disciplines.

## Introduction

The 50-meter freestyle swimming race is a test of explosive strength endurance, characterized by its short duration (men's record: 20.91 seconds; women's record: 23.67 seconds). Due to the brief time span of the race, glycolytic and phosphagenic metabolic pathways dominate, making upper body strength a critical factor [1]. This event can be performed using any of the four strokes: butterfly, backstroke, breaststroke, or freestyle. However, swimmers typically choose freestyle as it is the fastest and most efficient stroke [2].

Pull-ups are a bodyweight exercise that enhance upper body traction strength, performed in a closed kinetic chain where the terminal segment remains fixed. They can be executed with various grips, either pronated or supinated, which influence the muscle groups engaged. The primary muscles involved in this exercise include the latissimus dorsi, teres major and minor, trapezius, biceps, brachialis, deltoid, and pectoral muscles. A pronated grip increases activation of the latissimus dorsi, while a supinated grip engages the brachialis more intensely [3]. Thus, pronated grip pull-ups closely resemble the muscle engagement required in the 50-meter freestyle swimming event, with the latissimus dorsi being the principal muscle used in both exercises [4].

Pronated grip pull-ups have a greater biomechanical transfer to the freestyle stroke [5], suggesting that they could serve as both a predictor of swimming performance and a tool for training adaptations [6]. Previous research has noted the impact of upper body strength on swimming performance. Sharp and Troup (1982) found that greater upper body strength enhances performance in short freestyle events [7]. Storck (2017) observed a strong correlation between pull-ups and swimming performance, particularly in events longer than 400 meters [8]. Storck emphasized the importance of incorporating upper body strength exercises in training, highlighting the significant role of the latissimus dorsi for swimmers. Similarly, Pérez et al. (2018) confirmed a positive correlation between pull-up mechanics and performance in short swimming events, underscoring the importance of upper body strength in this sport [9].

Despite the recognized importance of strength in explosive endurance events and the potential benefits of pull-up exercises, there is inconsistency in the literature due to varied study methods and limited research on the effect of pull-ups on swimmers. Given the similarities between pull-ups and freestyle swimming, this study aims to observe the effect of pull-up training on 50-meter freestyle swimming performance.

## Materials and methods

Participants

The study included eight competitive swimmers (37.5% women) with an average age of 21 ± 6.7 years, average height of 178 ± 5.3 cm, and average body mass of 73 ± 7.0 kg. Swimmers were selected based on the following criteria: a) membership in the competitive section of the team; b) commitment to competition and training; c) attendance at pool training at least three times a week; d) attendance at strength training at least twice a week; e) for male swimmers, a 50-meter freestyle time of less than 26 seconds; f) for female swimmers, a 50-meter freestyle time of less than 30 seconds; and g) no injuries or discomfort in the six months prior to the study that would hinder regular training. All swimmers met the inclusion criteria and participated voluntarily after signing written informed consent forms.

The swimming pool tests were conducted at Piscinas Almería in the city of Huesca, located at Rda. Misericordia, s/n, 22001 Huesca. Then, the pull-up test and pull training were carried out in the gymnasium of Pabellón Deportivo Río Isuela, also located at Rda. Misericordia, s/n, 22001 Huesca. The study adhered to the Helsinki guidelines and received approval from the Ethics Committee of Comité de Ética de la Investigación de la Comunidad de Aragón (CEICA, Research Ethics Committee of the Community of Aragon) Aragón (No. PI23-161).

Intervention

The intervention lasted seven weeks, during which pull-up exercises were incorporated into the swimmers' bi-weekly strength training sessions. Pool training routines remained unchanged, and swimmers continued their regular training schedules. Performance was assessed at the beginning and end of the study through four tests: two evaluating swimming performance and two assessing upper body strength via pull-up exercises.

In the first week, the upper body strength tests were conducted first, both on the same day, followed by the swimming tests 48 hours later, also on the same day. This procedure was repeated in the final week to evaluate the impact of pull-up training on 50-meter freestyle performance and to determine if pull-up exercises could predict performance in this event.

Pull-up test

Two pull-up tests were conducted to measure different strength parameters [10]. Prior to these measurements, swimmers were instructed and allowed to practice the tests to become familiar with the movements. The participants refrained from any strength training for 48 hours before the tests to avoid fatigue, although they continued their regular pool training. In the first test, participants performed three pull-ups at maximum speed and power. In the second test, they performed as many pull-ups as possible within 30 seconds.

For both tests, swimmers started by hanging from the bar without touching the ground, using a pronated grip, with their arms fully extended. When the subjects were in this position and without any swinging, a signal was given to start. In the first test, swimmers were required to perform the pull-ups without pausing between repetitions to maintain maximum speed and power, with each pull-up only counting if their chin cleared the bar. In the second test, a timer started with the signal, and time updates were given every five seconds. Similarly, each pull-up only counted if their chin cleared the bar, but swimmers were allowed to rest between pull-ups. This rest had to be in the initial hanging position, with arms extended, without swinging, and without touching the ground.

Performance in both tests was recorded using a Vitruve linear encoder (Speed4Lift, Madrid, Spain), directly connected to an iOS device, allowing real-time monitoring of all variables of interest [11]. This device is considered the gold standard for measuring speed in specific strength exercises. It was placed on the floor directly beneath the subject and attached to their body with a belt. Pull-ups had to be performed without any swinging to prevent erroneous data. If the subject swung, the data from that pull-up were discarded. The iOS connection enabled instant feedback to the athlete. During the 30-second test, if a pull-up did not clear the bar, the encoder indicated a shorter range of motion. If this happened repeatedly, the subject was required to stop before the 30 seconds ended. However, if it occurred randomly, the specific pull-up was discarded and not included in the final result.

When explosive strength was measured using the Vitruve device, data for each pull-up included the maximum execution speed (m/s), average execution speed (m/s), average power (W), and 1RM. For strength endurance, data for each pull-up included the maximum execution speed (m/s), average execution speed (m/s), average power (W), and the maximum number of pull-ups in 30 seconds.

Prior to the tests, all subjects followed the same warm-up routine (both in the initial and final measurements), which began with shoulder, knee, and hip mobility exercises, light sets (3-4 x 10-15 reps) of shoulder flexion, extension, abduction, and adduction exercises with bands, and progressively more difficult push-ups. Then, they performed two sets of three assisted pull-ups with bands at maximum speed. After a five-minute rest, the first test of three pull-ups at maximum speed was conducted. Following a 15-minute rest, the second test of performing as many pull-ups as possible in 30 seconds was conducted.

Pool test

Two pool tests were conducted to measure various performance parameters.

First Test: Sprint Start to 15 Meters

The participants started from within the pool and performed a sprint start to 15 meters at maximum speed. They began in a streamlined position (horizontal, pronated, and not touching the pool floor) with feet near the wall, head underwater, and body still. At the signal, they broke through the water's resistance and swam freestyle at a maximum speed to 15 meters. If a participant pushed off the wall or started before the signal, they had to repeat the test after a 15-minute rest.

Second Test: 50-Meter Freestyle

The participants performed the 50-meter freestyle at maximum speed in a 25-meter pool. They started from the diving block with a head start. If any participant started before the signal, the test was repeated after a 15-minute rest. Performance in the first test was measured with a stopwatch, recording the final time when the participant's head passed the 20-meter mark. In the second test, performance was measured with a FINIS Pace Clock 3 x 100 M stopwatch (FINIS, Inc., California, United States), recording three times: the split time for the first 25 meters (when the swimmer touched the wall with their feet during the turn), the split time for the second 25 meters (calculated by subtracting the final time from the first split time), and the final time when the swimmer touched the wall with their hand.

The pool tests were conducted 48 hours after the pull-up tests to ensure the swimmers were not fatigued. All participants followed the same warm-up routine, similar to their pre-competition routine. This included mobility exercises out of the water for the knees, hips, and shoulders, followed by four shoulder injury prevention exercises (3 x 10 repetitions of each). The in-water warm-up consisted of 500 meters of freestyle, eight sets of 50 meters combining technique drills and kicking, two sets of 50 meters with varying speeds, and four to five maximal speed starts from the block to 12.5 meters. The first test, the 20-meter sprint start, was performed first, followed by 12 minutes of active rest, during which swimmers did about 300 meters of easy swimming, four sets of 100 meters with progressively increasing speed without ending at a high speed, and 100 meters of easy swimming. The second test, the 50-meter freestyle at maximum speed from the block, was then conducted. Swimmers were not given prior instructions for these tests as they were accustomed to performing these exercises.

Both the pull-up and swim tests were conducted in the same manner during the initial week of the intervention and the final week of the study, under the same conditions, warm-up routine, rest periods, and test order.

Pool training

**Figure 1:**
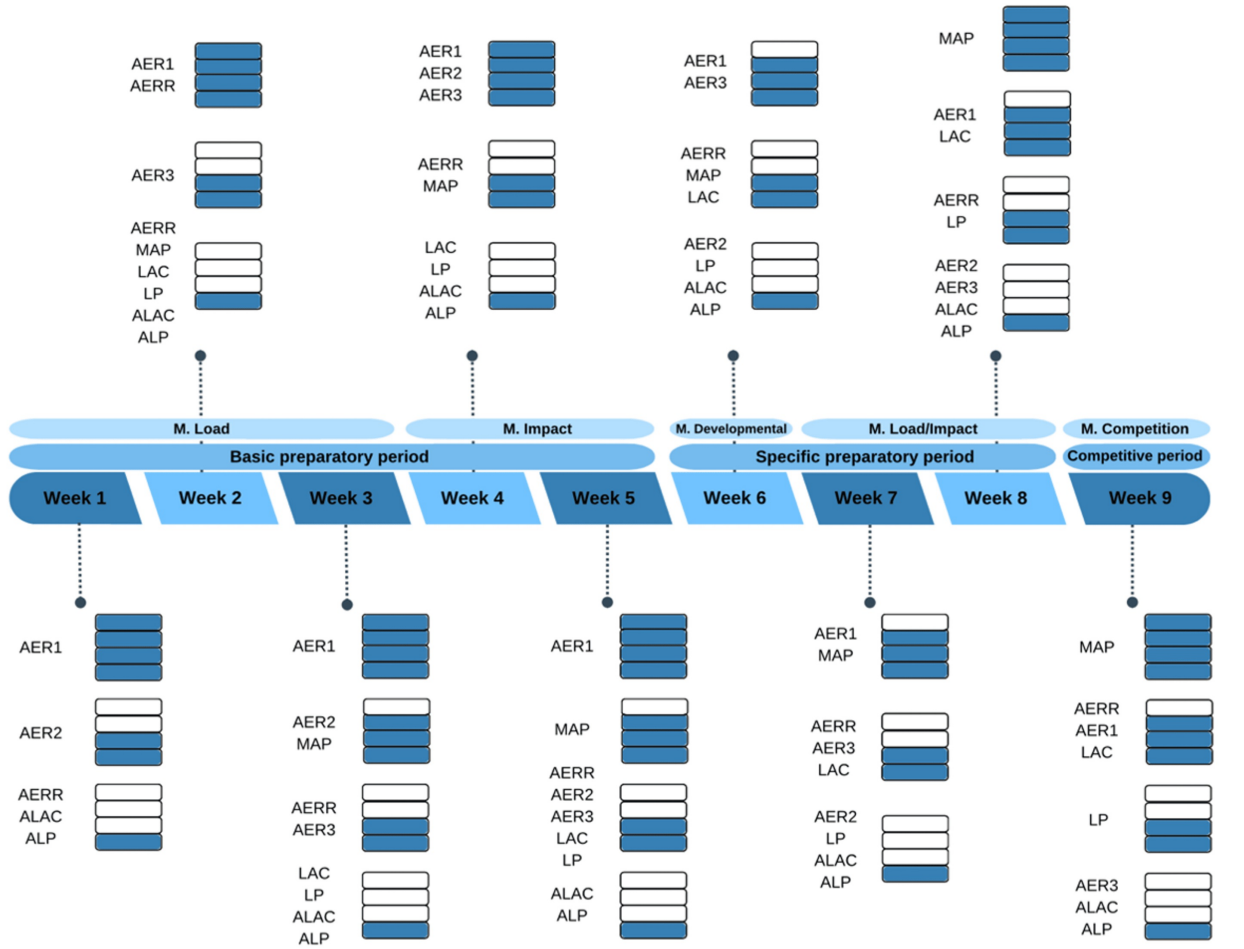
Representation of the swimming season plan with performance factors AERR, aerobic recovery; AER1, up to lactate threshold; AER2, up to critical speed/anaerobic threshold; AER3, up to maximum pace that achieves VO2max; MAP, maximum aerobic power; LAC, lactic capacity; LP, lactic power; ALAC, alactic capacity; ALP, alactic power

The pool training sessions remained unchanged; swimmers adhered to their scheduled seasonal training plan. This plan included five to six weekly sessions, each lasting one hour and 45 minutes, totaling approximately 10 hours per week (Figure 1). The figure outlines the swimming training schedule during the intervention period, detailing the performance factors emphasized in each session. Factors emphasized more frequently are indicated with four levels of blue shading, while those emphasized less frequently are marked with one level of blue shading.

Pull-up training

**Figure 2:**
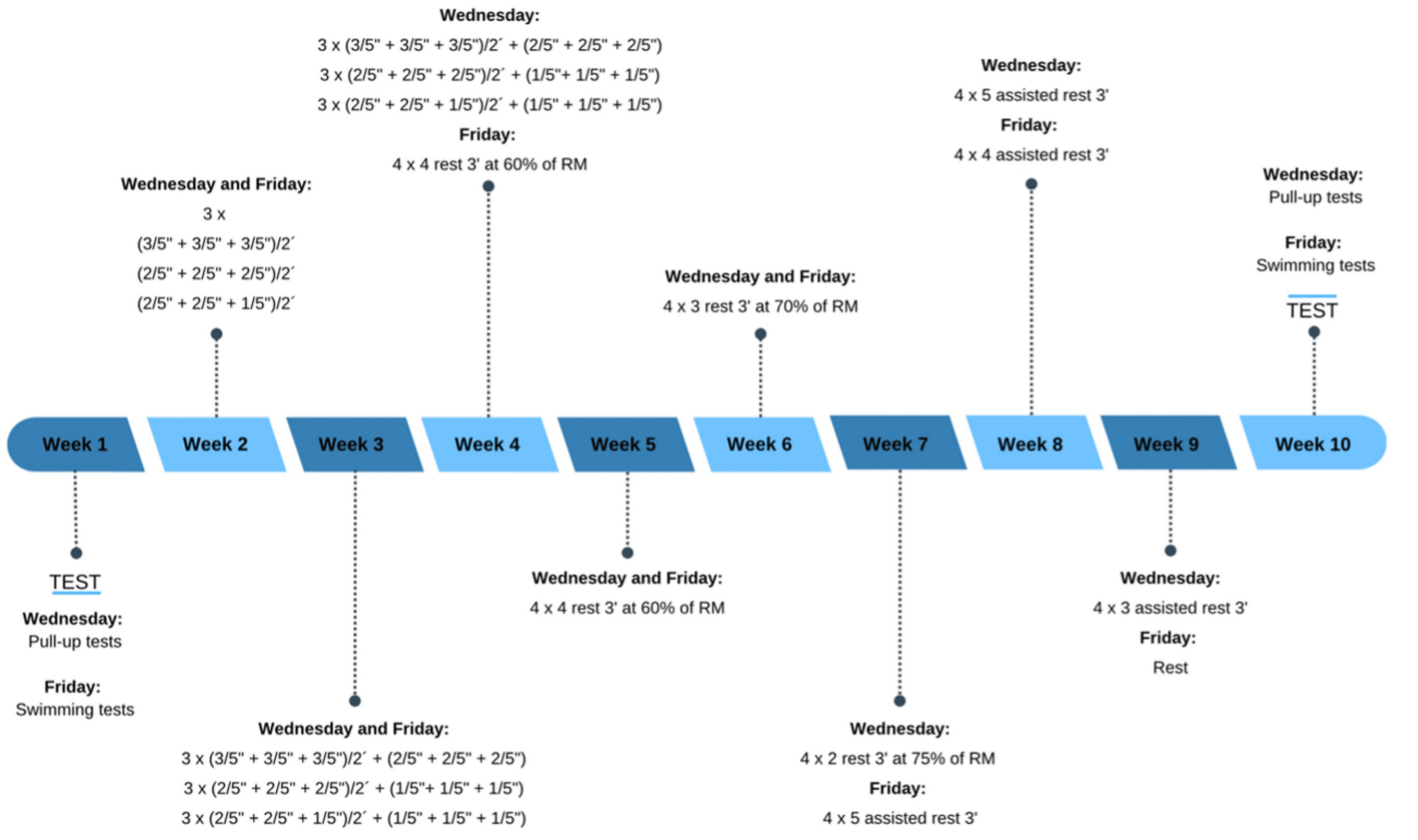
Representation of the plan followed during the intervention, in strength training, regarding the pull-up exercise.

During the intervention, pull-up exercises were integrated into the swimmers' regular strength training sessions, which were conducted twice a week for a total of two hours per week (one hour on Wednesdays and one hour on Fridays). All subjects had prior experience with pull-up training, ensuring the correct execution of the exercise. Furthermore, all training sessions were supervised by the strength and conditioning trainer, thereby ensuring the correct execution of the exercise. The pull-up and swimming tests were administered under consistent conditions at both the beginning and end of the intervention, facilitating a reliable comparison of the data obtained from both tests. Throughout the intervention, the focus was on achieving the maximum execution speed. Therefore, training primarily utilized body weight instead of very heavy loads relative to the swimmers' one-repetition maximum (RM) (Figure 2).

In these sessions, the participants also continued their regular physical training according to the planned seasonal schedule. The primary exercises performed included deadlifts, bench presses, full squats, and core work. In addition, secondary exercises such as Russian kettlebell swings and shoulder stability exercises were incorporated. Swimmers in the absolute category performed these exercises in blocks of 2-4-1 sets of 8-5-3 repetitions, with an effort level of 16-8-5 as the cycle progressed. The focus was always on achieving maximum speed, power, and explosive strength. Therefore, the weights used during the cycle ranged from 25% to 75% of their one-repetition maximum (1RM), aiming to maximize the force applied within the shortest unit of time [12]. Similarly, swimmers in the junior and infant categories performed these same exercises in blocks of 2-4-2 sets of 8-6 repetitions, with an effort level of 20-15-12 as the cycle progressed. They also focused on maximum speed, power, and explosive strength.

Statistical analysis

Data were described as mean (standard deviation), median, interquartile range, and absolute range. Descriptive statistics and univariate data visualizations suggested that probability distributions were not Gaussian, and we proceeded with a non-parametric analysis. Accordingly, differences over time were assessed using Wilcoxon signed-rank tests with continuity corrections (V). In addition, effect sizes were reported using rank-biserial correlations (rrb), and the parameter uncertainty was calculated with the 95% confidence intervals (95% CI), estimated using the normal approximation (via Fisher's transformation). In this analysis, effect sizes were interpreted using established thresholds where an rrb value of 0.1 was considered to represent a small effect, an rrb value of 0.3 was interpreted as indicating a medium effect, and an rrb value of 0.5 or greater was deemed to signify a large effect. Data analyses were performed in R, and statistical significance was assumed when p < 0.05.

## Results

Upper body strength and endurance

The three pull-up test results suggest various changes in the upper-body explosive strength. The mean power showed a statistically significant increase from week 1 to week 9 (p = 0.016), with a large effect size. However, no significant changes were observed in the maximum execution speed (p = 0.33) or average execution speed (p = 0.12), with medium effect sizes. In addition, the estimated 1RM did not show significant changes either (p = 0.38), with a small effect size.

**Figure 3:**
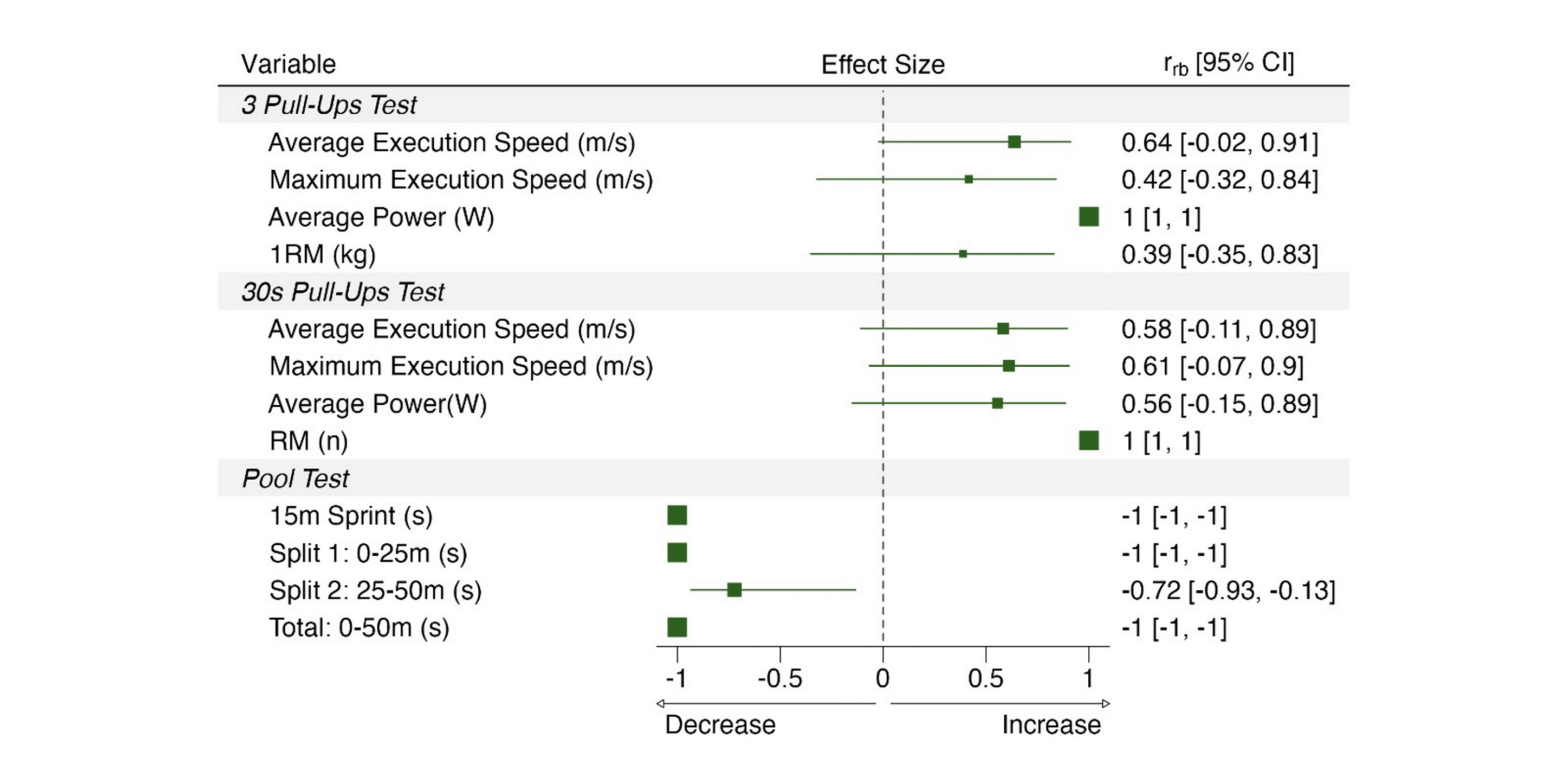
Effect size for the differences over time.

In the 30-second pull-up test, the estimated 1RM improved significantly from week 1 to week 9 (p = 0.014), with a large effect size. The mean power did not change significantly (p = 0.20), with a medium effect size. Similarly, no significant improvements were observed in maximum execution speed (p = 0.15) or average execution speed (p = 0.16), with medium effect sizes. Performance results are described in Table 1, and effect sizes for time changes can be inspected in Figure 3.

Swimming performance

**Figure 4:**
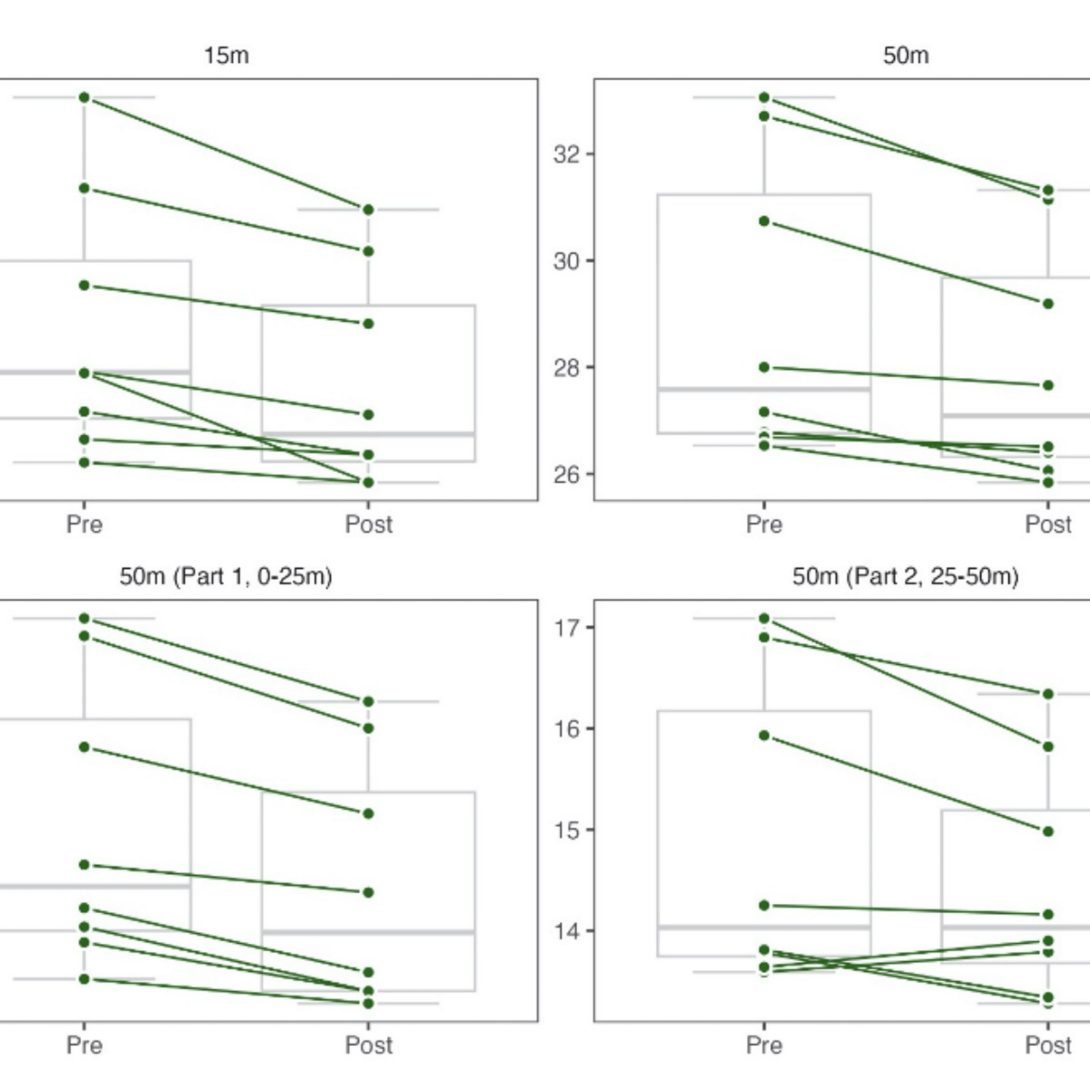
Performance evolution during the 15- and 50-meter swimming trials.

The 15-meter sprint test results showed significant improvements in swimming explosive strength. The sprint time decreased significantly from week 1 to week 9 (p = 0.014), with a large effect size. Figure 4 provides a detailed view of individual performance changes in swimming trials.

The total time for the 50-meter swim was significantly reduced (p = 0.008), with a large effect size. The first 25-meter segment of the 50-meter swim also showed a significant decrease in time (p = 0.014), with a large effect size. However, the second 25-meter segment did not show a statistically significant change (p = 0.078), although the effect size was still large.

## Discussion

This study investigated the impact of integrating pull-up exercises into the training routines of competitive 50-meter freestyle swimmers. The main findings were as follows: a) the pull-up training resulted in improvements in the 15-meter sprint test, and b) improvements in the 50-meter freestyle test were primarily influenced by the first 25 meters.

Strength training programs are common among swimmers, although their general benefits are commonly questioned in the literature, as many coaches believe they conventional strength training may lead to hypertrophy or reduced flexibility, potentially increasing drag forces and thus decreasing swimmer performance [13]. Contrary to that, previous evidence shows that improving power is one of the most crucial components of a strength program for swimmers [14], especially when the chosen exercises transfer to the sport itself, involving movement patterns relevant to swimming [15].

The current study reported an increase in speed in the 15-meter and 50-meter freestyle tests, the latter primarily influenced by the first 25 meters, which also led to a decrease in the total time of the test, mainly due to reduced time in the first half of the race. The swimmers in this study were at the beginning of their second macrocycle of training, which would guarantee the specificity of the improvements achieved, as they had previously completed a full training cycle. With this test, the aim was to reduce the significance of the lower limbs to better observe the influence of the upper limbs, which theoretically were affected by pull-up training. The strength and power of the lower limbs decisively influence performance in speed events, as starting from the blocks and the turning phase can cover up to 30% of the total distance in the race [16-18]. However, previous evidence also suggests the importance of upper limbs in sprint freestyle, in which performance would primarily depend on stroke length [19]. The findings of the current study highlight the potential positive impact of pull-up training on upper-limb power and speed swimming performance.

The potential of upper limb strength exercises has been discussed in previous scientific literature. For instance, Morouço et al. (2011) observed that dry-land strength and power training exercises such as lat pulldowns, bench presses, or medicine ball throws positively correlated with swimming power, with these exercises being predictors of performance [6]. This finding was similar to the study by Pérez et al. (2018), where they highlighted the importance of back muscle strength and endurance in swimming during 30-second freestyle tests [10]. In another study akin to the aforementioned ones, Storck (2017) found a strong correlation between pull-ups and swimming performance compared to lat pulldowns and bench presses [8]. This was attributed to pull-ups being more specific and closely resembling the movements involved in freestyle and butterfly strokes. Previous literature has shown that dry-land training improves strength and power [6] and reduces muscle imbalances typical in swimming [20]. Based on the findings of the current and previous studies, including dry-land strength and power training alongside swimming sessions is crucial for enhancing performance.

Despite these positive outcomes, the study faced several limitations. The small sample size together with age and gender disparity may have influenced the results. Despite the participation of both men and women in the study, no gender disparity was found. The data revealed that the improvements observed helped both men and women similarly. This reflects the difficulties commonly found in sports science interventional studies attempting to assess the outcomes during specific training strategies that may affect negatively the competitive season. Furthermore, the concurrent pool training schedules and individual daily habits of participants could have influenced performance outcomes. Future research should aim to address the study's limitations by employing larger, more homogeneous samples and controlling for variables like age, gender, and training schedules more rigorously. This includes examining gender disparities and the specific improvements that could be obtained by differentiating between male and female participants. As this is a pilot study, expanding the sample size and introducing a control group are essential steps for future research. These efforts could provide clearer insights into the effectiveness of pull-up exercises in optimizing swimming performance, particularly in sprint disciplines.

## Conclusions

This study provides valuable insights into the effectiveness of integrating pull-up exercises into the training regimens of competitive swimmers. The findings highlight significant improvements in key performance metrics, including enhanced sprint start speed and increased velocity during the initial phase of the 50-meter freestyle swim. These improvements directly contributed to reduced overall race times, underscoring the importance of upper body strength and power in swimming performance.
